# Analysis of Sequence Polymorphism and Population Structure of *Tomato chlorotic dwarf viroid* and *Potato spindle tuber viroid* in Viroid-Infected Tomato Plants

**DOI:** 10.3390/v4060940

**Published:** 2012-06-05

**Authors:** Xianzhou Nie

**Affiliations:** Potato Research Centre, Agriculture and Agri-Food Canada, P.O. Box 20280, 850 Lincoln Road, Fredericton, New Brunswick, E3B 4Z7, Canada; Email: xianzhou.nie@agr.gc.ca; Tel.: +1-506-4524843; Fax: +1-506-452-3260

**Keywords:** TCDVd, PSTVd, sequence variants, polymorphism, population

## Abstract

The sequence polymorphism and population structure of *Tomato chlorotic dwarf viroid* (TCDVd) (isolate Trust) and *Potato tuber spindle viroid* (PSTVd) (isolate FN) in tomato plants were investigated. Of the 9 and 35 TCDVd clones sequenced from 2 different TCDVd-infected plants, 2 and 4 sequence variants were identified, respectively, leading to a total of 4 sequence variants of 360 nucleotides in length. Variant I was identical to AF162131, the first TCDVd sequence to be reported, and the rest exhibited 1 to 3 nucleotide differences, all in the T_R_ domain, from AF162131/variant I. Of the 33 and 29 PSTVd clones sequenced from 2 different PSTVd-infected plants, 8 and 9 sequence variants were found, respectively, leading to a total of 15 variants ranging in length from 356 to 359 nucleotides. The variant I was identical to EF044303, a PSTVd reported in Russia. The rest exhibited 1 to 11 nucleotide differences scattering in all five domains from EF044303/variant I. The results demonstrated for the first time that TCDVd, like many other viroids including PSTVd, exists in host plants as a collective group comprised of various sequence variants. However, in comparison to PSTVd, TCDVd is less polymorphic in tomato plants as fewer variants and lower haplotype/nucleotide diversities were observed.

## 1. Introduction

Viroids are the smallest plant pathogens that can cause a wide range of symptoms from symptomless to severe stunting, leaf/stem necrosis and foliar/fruit deformation, depending on host plant and viroid species [1]. Viroids consist of a single-stranded, non-protein-coding, covalently closed RNA of 239-401 nucleotides (nt) [2]. Currently, 29 viroid species belonging to two families, *Pospiviroidae* and the *Avsunviroidae*, have been recognized [2]. Most viroids characterized so far are grouped in the *Pospiviroidae* family. They contain five structural domains/regions [3]: left terminal region (T_L_), pathogenic region (P), central conserved region (C), variable region (V) and right terminal region (T_R_).

*Potato spindle tuber viroid* (PSTVd), the type member of genus *Pospiviroid*, family *Pospiviroidae*, was the first viroid species to be identified [4]. It infects potato, tomato and many other solanaceous and non-solanaceous plant species and causes significant yield loss and quality degradation in potato and tomato crops [5,6,7,8]. Although eradicated from potato production systems in many countries [9], the pathogen has recently occurred in isolated circumstances in greenhouse tomato crops [8,10,11,12]. In addition to PSTVd, *Tomato chlorotic dwarf viroid* (TCDVd) [6], a closely related species to PSTVd, has also been widely reported in greenhouse tomato crops worldwide [6,8,13,14,15].

Due to the lack of protein-coding capacity, viroids rely on their hosts for replication. Members of the *Pospiviroidae* family replicate in the nucleus by host DNA-dependent RNA polymerase II [1]. The lack of proof-reading activity of the plant polymerase(s), the large population size, and the rapid rate of RNA replication have been suggested to result in genetic polymorphism, as demonstrated in various viroids including PSTVd [16,17,18], *Citric exocortis viroid* (CEVd) [19,20], *Hop stunt viroid* (HSVd) [21], *Australian grapevine viroid* (AGVd) [22], *Pear blister canker viroid* [23], *Peach latent mosaic viroid* (PLMVd) [24,25], *Grapevine yellow speckle viroid-*1 (GYSVd-1) [26], and *Chrysanthemum stunt viroid* (CSVd) [27]. In this study, the genetic variants of TCDVd and PSTVd in tomato plants were investigated and analyzed. The results revealed the polymorphic nature of TCDVd in host plants. However, the TCDVd isolate was less polymorphic than the PSTVd isolate.

## 2. Results and Discussion

**Figure 1:**
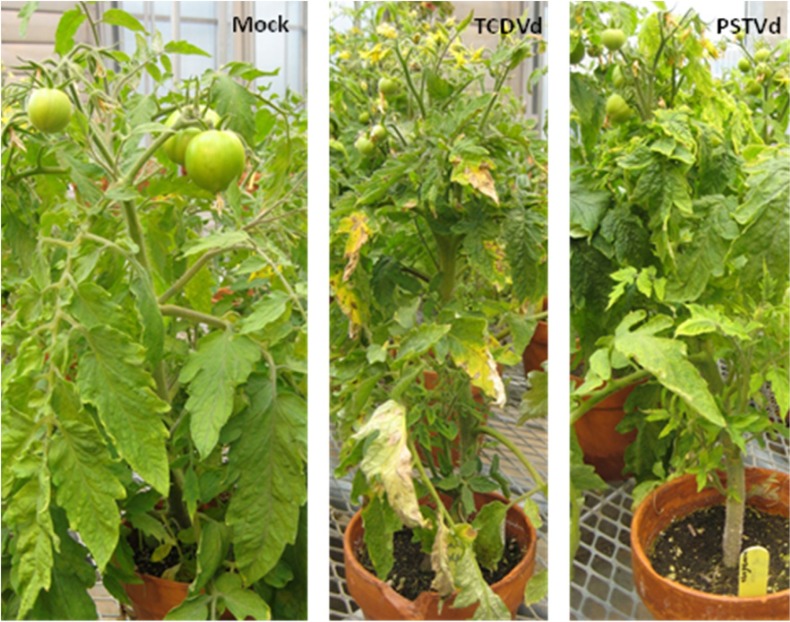
Symptoms induced by Tomato chlorotic dwarf viroid (TCDVd) isolate Trust and *Potato spindle tuber viroid* (PSTVd) isolate FN in tomato plants. Tomato (cultivar Sheyenne) plants at the four-leaf stage were mechanically inoculated with TCDVd or PSTVd (leaf extract from an infected tomato plant) or buffer (Mock) and grown in the greenhouse. Pictures shown were taken 45 days after inoculation.

Typical viroid-induced symptoms including stunting, bunching, leaf deformation, leaf and fruit size reduction, and leaf chlorosis were observed in tomato (cultivar Sheyenne) plants infected with TCDVd or PSTVd (Figure 1), similar to that reported previously [2,6,28,29,30]. In potato (cultivar Shepody), both viroids induced growth stunting, foliar epinasty and spindle-shaped tubers (data not shown). The TCDVd isolate (designated TCDVd Trust in this paper) was previously characterized and sequenced [6], and one sequence (accession number AF162131) was reported. The PSTVd isolate (designated PSTVd FN in this paper), on the other hand, has not been molecularly characterized to date.

Many viroids, including PSTVd, have been suggested to propagate in their hosts as a population of similar but not identical sequences [16]. To reveal whether this is also true for TCDVd, and more specifically, to reveal whether there is more than one sequence variant in TCDVd isolate Trust, multiple full length cDNA clones generated from the RNA of an infected tomato plant were sequenced together with clones derived from a PSTVd-infected tomato plant. As shown in Table 1 and Table 2, sequence variants were detected in both viroids. For TCDVd, an initial sequencing of 9 clones from plant 1 detected 2 sequence variants at occurrence frequencies of 66.7% and 33.3%, respectively. Further sequencing of 35 clones obtained from plant 2 detected 4 variants at occurrence frequencies of 65.7%, 28.6%, 2.9% and 2.9%, respectively (Table 1). For PSTVd, 8 sequence variants at occurrence frequencies ranging from 3.0% to 30.3% were found in clones obtained from PSTVd-infected plant 1; and 9 variants at occurrence frequencies of 3.4% to 44.8% in clones obtained from plant 2 (Table 2). These results demonstrate that TCDVd, like PSTVd as well as many other viroid species [19,21,22,23,24,25,26], exists as a collective group comprised of various sequence variants. Nevertheless, TCDVd was less diverse than PSTVd in tomato plants, as fewer TCDVd variants and lower haplotype diversity (0.500 and 0.499 for TCDVd *vs.* 0.799 and 0.697 for PSTVd) was observed in the clones from the plants (Table 1 and Table 2).

All TCDVd variants were comprised of 360 nt. BLAST analysis against GenBank revealed that TCDVd variant I showed 100% sequence identity with AF162131, the very first TCDVd sequence to be reported [6], whereas the other variants exhibited varying degrees of differences with the TCDVd sequences deposited in the database (data not shown). Variant I was the dominant variant with an occurring frequency of 66.7% and 65.7% in plants 1 and 2, respectively, followed by variant II (33.3% in plant 1 and 28.6% in plant 2), and variants III and IV (0 in plant 1 and 2.9% for each in plant 2) (Table 1). Variant I differed from variants III and IV by 1 nucleotide at position in the reference sequence nt 165 (U *vs.* C) and nt 149 (A *vs.* G), respectively, and variant II by 3 nucleotides at nt 178 (U *vs.* A), 182 (C *vs.* U) and 183 (A *vs.* U). It is noteworthy that the isolate investigated in this study was the same isolate in which AF162131 was obtained [6], suggesting that variant I likely remained the predominant variant in the isolate ever since its discovery from tomato in the end of the 1990s. This discovery is consistent with the observation that predominant genotypes of pospiviroids are highly stable [31].

**Table 1:** *Tomato chlorotic dwarf viroid* (TCDVd) sequence variant population structure, occurrence frequency and nucleotide variation in TCDVd isolate Trust-infected tomato plants.

Sequence variant	Size (nt)	Occurrence frequency (%)		Nucleotide difference from the reference sequence ^a^
		Plant 1	Plant 2	
AF162131	360	n/a	n/a	n/a
I	360	66.7	65.7	No variation
II	360	33.3	28.6	178 (U to A); 182 (C to U); 183 (A to U)
III	360	0	2.9	165 (U to C)
IV	360	0	2.9	149 (A to G)
Haplotype diversity ^b^		0.500 ± 0.128	0.499 ± 0.071	
Nucleotide diversity ^c^		0.00417 ± 0.00107	0.00382 ± 0.00057	

^a^ AF162131 was used as a reference sequence. Nucleotide differences in each variant were indicated in relation to the reference sequence at positions in the reference; ^b/c^ Haplotype and nucleotide diversities were calculated using the software DnaSP v5; n/a: not applicable.

PSTVd variants consisted of 356-359 nucleotides (Table 2), and were much more diverse than TCDVd variants. One variant, which was designated variant I, showed 100% sequence identity with EF044303, a sequence obtained from the potato PSTVd isolate “Bugry-97” from Russia [32]. The rest exhibited 1 to 11 nucleotide differences from variant I/EF044303 (Table 2). Variants II to VI, IX and X differed from variant I by 1-2 nucleotides; variants XI and XII differed from variant I by 3 and 4 nucleotides, respectively; whereas variant VII, VIII, XIII to XV differed from variant I by 7-11 nucleotides. No clear-cut predominant PSTVd variants were observed in plant 1 and several variants occurred at a frequency higher than 15%: variant VII occurred at the highest frequency of 30.3%, followed by variant III (27.3%), I and II (15.2% for each). In plant 2, variant I was the most abundant variant, occurring at a frequency of 44.9%, followed by XV (20.7%), II/XIII/XIV (6.9% for each), and IX/X/XI/XII (3.4% for each). Clearly, PSTVd not only consisted of more diverse sequence variants than TCDVd, but also exhibited higher nucleotide diversities than the latter (0.0113 and 0.00944 for PSTVd *vs.* 0.00417 and 0.00382 for TCDVd) (Table 1 and Table 2). Interestingly, the PSTVd isolate studied in this research was not related to the Russia isolate “Bugry-97” from which EF044303 was obtained [33], suggesting that variant I was likely an “ancestor” variant from which other variants might have been derived. It is unknown what might have caused the difference in variant population composition between the two PSTVd-infected plants as the two plants were inoculated from a same inoculum.

**Table 2:** *Potato spindle tuber viroid* (PSTVd) sequence variant population structure, occurrence frequency and nucleotide variation in PSTVd isolate FN-infected tomato plants.

Sequence variant	Size (nt)	Occurrence frequency (%)		Nucleotide difference from the reference sequence ^a^
		Plant 1	Plant 2	
EF044303	358	n/a	n/a	n/a
I	358	15.2	44.8	No variation
II	358	15.2	6.9	120 (C to U)
III	357	3.0	0	120 (C to U), 236 (C to deletion)
IV	358	3.0	0	18 (U to C), 120 (C to U)
V	358	3.0	0	40 (U to A), 120 (C to U)
VI	358	27.3	0	120 (C to U), 253 (G to A)
VII	357	30.3	0	120 (C to U), 127 (G to A), between 145 and 146 (insertion of C), 200 (G to U), 211 (C to deletion), 212 (G to A), 232 (C to deletion), 255 (C to U), 306 (C to U), 307 (U to C), 310 (C to U)
VIII	356	3.0	0	200 (G to U), 211 (C to deletion), 212 (G to A), 232 (C to deletion), 255 (C to U), 306 (C to U), 307 (U to C), 310 ( C to U)
IX	357	0	3.4	277 (G to deletion)
X	359	0	3.4	Between 145 and 146 (insertion of C)
XI	358	0	3.4	173 (G to A), 306 (C to U), 307 (U to C), 310 (C to U)
XII	358	0	3.4	306 (C to U), 307 (U to C), 310 (C to U)
XIII	357	0	6.9	120 (C to U), 127 (G to A), between 145 and 146 (insertion of C), 200 (G to U), 211 (C to deletion), 212 (G to A), 232 (C to deletion), 343 (A to G)
XIV	357	0	6.9	120 (C to U), 127 (G to A), between 145 and 146 (insertion of C), 200 (G to U), 211 (C to deletion), 212 (G to A), 232 (C to deletion)
XV	357	0	20.7	120 (C to U), 127 (G to A), between 145 and 146 (insertion of C), 200 (G to U), 211 (C to deletion), 212 (G to A), 232 (C to deletion), 306 (C to U), 307 (U to C), 310 ( C to U)
Haplotype diversity ^b^		0.799 ± 0.034	0.697 ± 0.078	
Nucleotide diversity ^c^		0.01130 ± 0.00103	0.00944 ± 0.00115	

^a^ EF044303 was used as a reference sequence. Nucleotide differences in each variant were indicated in relation to the reference sequence at positions in the reference; ^b/c^ Haplotype and nucleotide diversities were calculated using the software DnaSP v5; n/a: not applicable.

**Figure 2:**
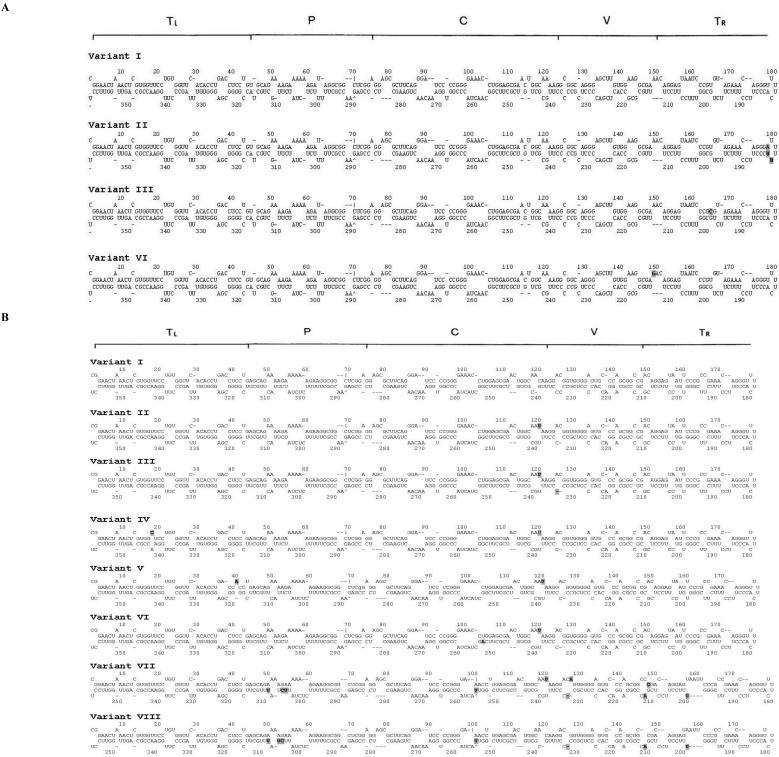
Secondary structure of *Tomato chlorotic dwarf viroid* (TCDVd) and *Potato spindle tuber viroid* (PSTVd) sequence variants. (**A**) TCDVd; (**B**) PSTVd variants I to VIII. T_L_: left terminal region, P: pathogenic region, C: central conserved region, V: variable region, T_R_: right terminal region. The shaded nucleotides are nucleotides that were different from variant I, which is identical to the TCDVd sequence AF162131 (A) or the PSTVd sequence EF044303 (B).

Another major difference between TCDVd and PSTVd populations was the location of the nucleotide mutations/variations in viroid secondary structures despite the fact that the mutations did not alter the rod-shaped structures (Figure 2). All the variations/mutations in TCDVd occurred at the terminal right (T_R_) region (Figure 2A) whereas the variations/mutations in PSTVd population occurred at all five domains (Figure 2B), consistent with a previous report by Gόra-Sochacka *et al.* [17]. Although we cannot completely rule out that the single nucleotide differences among variants might be artificially generated during PCR by the *Taq* polymerase, the possibility is slim because of the short fragment length of the viroid. Moreover, the slight possibility of error does not alter the overall variant diversity difference between the two viroids.

**Figure 3:**
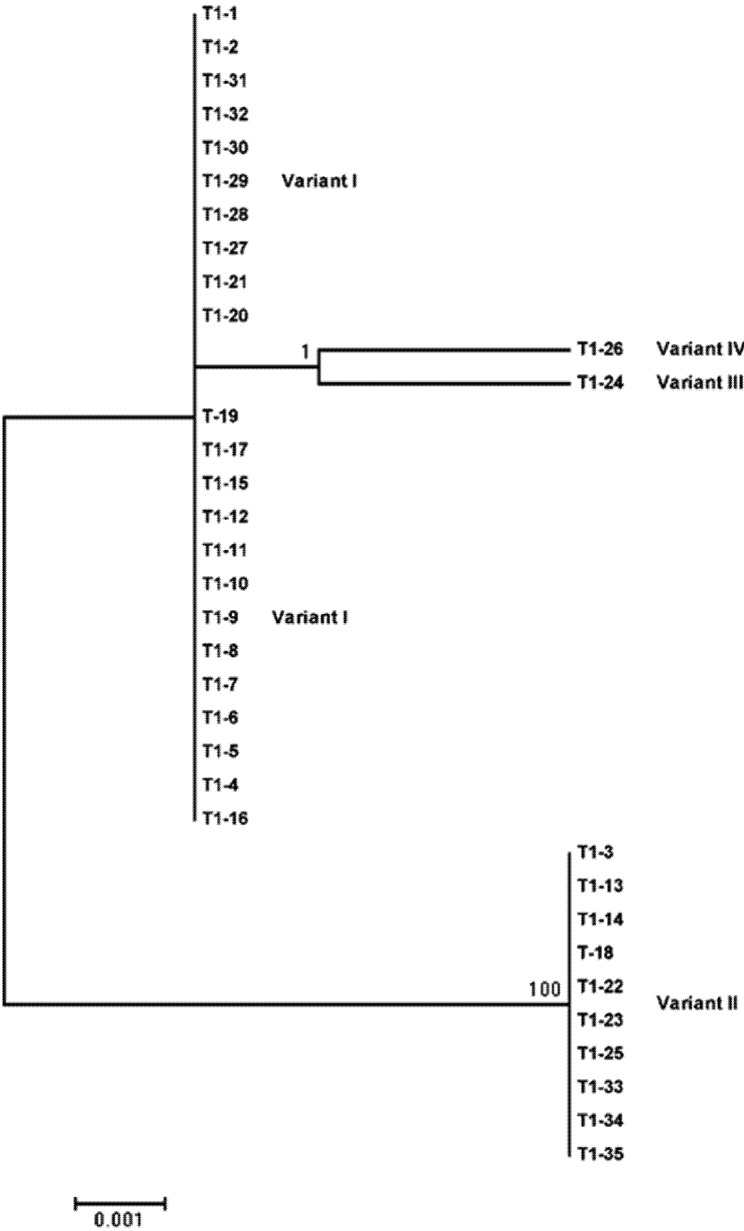
Phylogenetic analysis of *Tomato chlorotic dwarf viroid* (TCDVd) sequence variants. The evolutionary history was inferred by using the Maximum Likelihood method based on the Tamura-Nei model. The percentage of trees in which the associated taxa clustered together is shown above the branches. Initial tree(s) for the heuristic search were obtained automatically by applying Neighbor-Join and BioNJ algorithms to a matrix of pairwise distances estimated using the Maximum Composite Likelihood approach, and then selecting the topology with superior log likelihood value. The tree is drawn to scale, with branch lengths measured in the number of substitutions per site. Evolutionary analyses were conducted in MEGA5.

**Figure 4:**
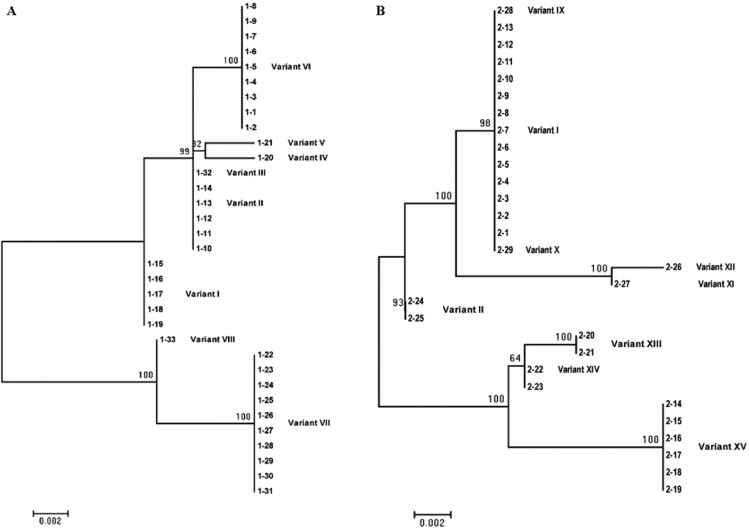
Phylogenetic analysis of *Potato spindle tuber viroid* (PSTVd) sequence variants. (**A**) Phylogenetic tree of PSTVd variants from plant one. (**B**) Phylogenetic tree of PSTVd variants from plant two. The analyses and tree constructions were conducted as described in Figure 3.

Despite the considerable differences between PSTVd and TCDVd in variant diversity, the two shared a similar trend in phylogeny. As shown in Figure 3 and Figure 4, two major branches were found for both viroids. For TCDVd, variants I, III and VI formed a cluster/branch, whereas variant II stood alone (Figure 3). For PSTVd in plant 1, variants I, II, III, IV, V and VI formed a branch whereas variants VII and VIII formed the other branch (Figure 4A). In plant 2, variants I, II, IX, X, XI and XII formed a branch, and VIII, XIV and XV formed the other branch (Figure 4B). A similar phylogenetic tree formed when PSTVd variants from both plants were combined (data not shown), confirming the existence of two population groups in both TCDVd and PSTVd. Nevertheless, the population composition of TCDVd was much simpler than that of PSTVd, further supporting the above analysis that the TCDVd isolate Trust was less polymorphic than the PSTVd isolate FN. Whether the relative simplicity of TCDVd in sequence diversity in isolate Trust was due to the higher stability of the predominant variant (*i.e.*, variant I) or due to other factors such as less mutagenic pressure [27] the viroid endured during its existence in the host plants is unknown. To answer these questions, further research is underway to investigate the diversification of major TCDVd and PSTVd variants in tomato plants using single cDNA clones of these variants.

## 3. Experimental Section

### 3.1. Viroid Sources

TCDVd isolate Trust was the same tomato isolate characterized and reported previously by Singh *et al.* [6]. The viroid was maintained in tomato cultivar Sheyenne plants in a greenhouse with 16 h light/day with a 16/8 h (light/day) cycle. The ambient light is supplemented with artificial light or shading to give a light intensity of 90 µm^2^/s. The temperature was 20–25 °C, and the humidity was 75%. PSTVd isolate FN was a potato isolate provided by A. Murphy (Potato Research Centre, Agriculture and Agri-Food Canada) and maintained in tomato cultivar Sheyenne plants. Both TCDVd Trust and PSTVd FN were tested for their pathogenicity in potato (cultivar Shepody) and tomato (cultivar Sheyenne) by infection via mechanical inoculation of healthy seedlings/plantlets with leaf exact from a TCDVd- or PSTVd-infected plant, and the resulting symptoms were observed and recorded. For the experiments on analysis of viroid sequence variants in tomato plants, tomato (Sheyenne) seedlings at the four-leaf stage were mechanically inoculated with leaf extract of a TCDVd- or PSTVd-infected tomato plant, and grown in the greenhouse under the above described conditions. Three leaves from an inoculated plant were collected 30 days after inoculation, and subjected to RNA extraction.

### 3.2. Total RNA Isolation and Reverse Transcription-Polymerase Chain Reaction (RT-PCR)

Total RNA was isolated from tomato leaves (2 g) as described previously [33]. The RNA was dissolved in 200 µL H_2_O and quantified using a NanoDrop 2000 c UV-Vis spectrophotometer (Thermo Scientific, Wilmington, DE, USA).

Primers P3/P4 [34] (P3, reverse, complementary to nucleotides 68-92 of PSTVd (accession number U23059): 5'-CCGGATCCCTGAAGCCGCTCCTCCGAGC-3’; P4, forward, containing nt 87 to 110: 5'-TCGGATCCCCGGGGAAACCTGGAGCG-3'. The underline indicates the overlapping sequence) were used to amplify full length cDNA of TCDVd and PSTVd. Two-step RT-PCR was performed as described previously [33], using Moloney murine leukemia virus-reverse transcriptase (MMLV‑RTase) (Gibco BRL, Gaithersburg, MD, USA) for RT and *Taq* DNA polymerase (PE Applied Biosystems, Foster City, CA, USA) for PCR. The PCR was performed on a MJ Research PTC 200 DNA Engine Thermocycler (MJ Research, Watertown, MA, USA) with different annealing temperature regime, *i.e.*, 62 °C for the first five cycles, 60 °C for the second five cycles, 58 °C for the following 10 cycles, and 55 °C for the last 10 cycles. Each cycle consisted of denaturation (92 °C, 30 s), primer annealing (30 s) and primer extension (72 °C, 90 s). After the 30 cycles, a final extension of 10 min at 72 °C was also included. The amplification was then analyzed by gel electrophoresis in a 1.5% agarose gel containing 1× GelRed (Biotium Inc., Hayward, CA, USA), visualized under UV illumination and photographed with an imaging system (Alpha Innotech IS1000, San Leandro, CA, USA). 

### 3.3. Complementary cDNA Cloning and Sequencing

The PCR products of each targeted viroid were ligated into a pDrive Cloning Vector (Qiagen, Valencia, CA, USA) according to the manufacturer’s instructions. The ligated plasmids were transformed into *E. coli* DH5-α competent cells (New England BioLabs, Ipswich, MA, USA). A portion of the transformation mixture was spread onto LB agar plates containing ampicillin (75 µg/mL), X-gal and IPTG according to the manufacturer’s instructions, and incubated at 37 °C overnight. White colonies were screened further by colony-PCR with P3/P4 primer pair under the PCR conditions described above, and positive colonies were transferred into 2 mL LB medium (with 75 µg/mL ampicillin) and incubated at 37 °C overnight. Plasmid was extracted from the bacterial cells using Qiagen Plasmid Mini Kit (Qiagen). Nine to 35 viroid cDNA clones resulted from single PCR-ligation-screening processes were sent to Robarts Research Institute (London, Ontario, Canada) for forward and reverse sequencing using the T7 promoter and SP6 promoter primers, respectively.

### 3.4. Phylogeny, Secondary Structure and Nucleotide Diversity Analysis

For phylogenetic analysis, viroid sequences were first aligned using the ClustalW2 multiple sequence alignment program (version 2.21) [35,36]. The aligned sequences served as input for phylogeny analysis using MEGA5 [37]. The evolutionary history was inferred using the Maximum Likelihood method based on the Tamura-Nei model [38]. The statistical significance of branching was estimated by performing 100 replications of bootstrap re‑sampling from the original data using SEQBOOT. 

Viroid nucleotide polymorphism (e.g., haplotype and nucleotide diversities) within a population was analyzed using DNA Sequence Polymorphism (DnaSP) version 5 [39,40]. Nucleotide diversity (or per-site heterozygosity, π) was calculated from the average number of pairwise sequence differences in the sample ([41], Equation 10.5: π = *n*/(*n* − 1)Σ*x_i_*x*_j_*π*_ij_*) and its standard deviation is the square root of the variance ([41], Equation 10.7). Haplotype (gene) diversity and its sampling variance were estimated according to equations 8.4 and 8.12 with 2n replaced by n [41].

Prediction of secondary structures of viroids was carried out using the WEB-based Mfold program Version 3.1 [42,43]. The structures were generated on the principle of the minimum energy folding of an RNA sequence [44].

## 4. Conclusions

This research revealed for the first time that TCDVd exists in tomato plants as a collective group comprised of various sequence variants, consistent with reports on other viroids including PSTVd in the family *Pospiviroidae*. However, TCDVd was less polymorphic than PSTVd in the investigated isolates as fewer TCDVd sequence variants and lower haplotype/nucleotide diversities were observed. All sequence variants of TCDVd and PSTVd exhibited a rod-shaped secondary structure, a typical conformation for all pospiviroids, suggesting that the rod-like secondary structure might be essential for a sequence variant to propagate/exist in the host plants.
